# An introduction to the TPSN model: a comprehensive approach to reducing the theory-practice gap in nursing

**DOI:** 10.1186/s12912-022-01030-w

**Published:** 2022-09-21

**Authors:** Maryam Namadi Vosoughi, Vahid Zamanzadeh, Leila Valizadeh, Akram Ghahramanian, Mojgan Lotfi, Farzaneh Bagheriyeh, Afsaneh Pourmollamirza

**Affiliations:** 1 Department of Medical Surgical Nursing, School of Nursing and Midwifery, Tabriz University of Medical Sciences, Tabriz, Iran; 2 Department of Pediatric Nursing, School of Nursing and Midwifery, Tabriz University of Medical Sciences, Tabriz, Iran; 3 Department of Medical Surgical Nursing, School of Nursing and Midwifery, Urmia University of Medical Sciences, Urmia, Iran; 4 School of Nursing and Midwifery, Tabriz University of Medical Sciences, Tabriz, Iran

**Keywords:** Model, Theory-practice gap, Nursing, Clinical education

## Abstract

**Background:**

There are still concerns about the effectiveness of clinical education models which are done with the aim of reducing the theoretical-practical gap in nursing. In this article, we intend to describe an innovative model to create an integration and structured relationship between educational and healthcare provider institutions. The basis of this work is the full-time presence of nursing teacher in the clinical settings and the development of their role to improve the education of students and nurses and the quality of nursing services.

**Methods:**

This was a participatory action research. This action research was implemented in four steps of problem identification, planning, action and reflection. Interviews, focus groups and observation were used for the qualitative part. Clinical Learning Environment Inventory (CLEI), Job Satisfaction in Nursing Instrument questionnaires and Patient Satisfaction with Nursing Care Quality Questionnaire were completed before and after the study. Qualitative content analysis, paired and independent t test were used for data analysis.

**Results:**

The academic-practice integration Model of TPSN is a dynamic and interactive model for accountability in nursing Discipline. Unlike the medical education model that includes patients, students, and physicians as the three points of a triangle, this model, which is shaped like a large triangle, places the person in need of care and treatment (patient, client, family, or society) in the center of the triangle, aiming to focus on the healthcare receiver. The model consists of three components (Mentoring component, Preceptorship component, and integrated clinical education component). Each of the components of this model alone will not be able to eliminate the ultimate goal of bridging the theory-practice gap.

**Conclusions:**

A new and innovative model was proposed to reduce the theory-practice gap in the present study. This model increases the collaboration between educational institutions and healthcare settings compared with the previous models. The TPSN model helps students, nurses, and nursing instructors integrate theoretical knowledge with clinical practice and act as professional nurses.

## Background

The gap between theory and practice is one of the most significant and fundamental challenges of nursing [1, 2]. The theory-practice gap has led to a crisis in nursing care and has provoked criticism against nursing services [3]. There are different definitions of the concept of theory and practice gap in nursing [4, 5]. In this study, the theory-practice gap refers to the distance between what is taught in the classroom and what the student nurses experience in the clinical area.

There is a significant difference between theoretical learning and clinical nursing services. Theoretical knowledge of nursing is the basis of practice, while the practical environment determines the conditions in which theoretical knowledge is applied [3, 6]. The mismatch between theory and practice in clinical settings leads to the lack of use of theoretical knowledge and adherence to traditional methods in practice. This prevents the development of theoretical sciences in nursing and leads to a decline in the quality of nursing care [5, 7]. This gap and the resulting problems disrupt the process of professional socialization and lead to a negative professional identity in nursing [8]. Moreover, the theory-practice gap has adverse effects on students [9]. In this situation, nursing students are not able to resolve the conflict between the expectations of nursing teachers and realities of their workplace. Therefore, the adverse physical and psychosocial effects, including feelings of helplessness, depression, insecurity and etc. and eventual withdrawal from their profession, occur in these students [10, 11].

Many studies highlight the theory and practice gap of nursing education around the world. Several international studies from the United States, the United Kingdom, Australia, and Canada report the reason for the gap between theory and practice is the lack of structural and defined connection between clinical settings and nursing schools as well as between nurse teachers and nurses. This leads to organizational divergence and lack of coordination between educational and health institutions [12] [13]. In Iran, lack of effective interaction between care institutions and colleges, have been reported as the main causes of the gap between theory and practice in nursing [13, 14].

Effective communication between educational and healthcare institutions requires two-way interaction [6]. In other words, nurses should be used as assistants in the clinical education process, and clinical instructors should be used to develop and improve the services of healthcare and educational institutes [2, 6, 15]. The collaboration between academic institutions (nursing schools) and healthcare institutions (teaching hospitals) through sharing capiticites reduces the theory-practice gap, increases nursing research and the development of evidence-based nursing, improves patient safety and outcomes, creates opportunities for human resource development, facilitates clinical teaching, and creates a supportive learning environment [16, 17].

### Perceived problems with older clinical teaching models

A review of the literature shows that over the years, various methods have been developed to reduce the gap between theory and practice [3]. The first category is called collaborative clinical teaching [18] in which specific clinical nurses are involved in students’ clinical teaching. This approach and its methods are mainly designed to compensate for the gap in nursing teachers’ procedural skills caused by the large scope of educational settings and the teacher’s distancing from clinical settings [19].

According to the literature, the main shortcoming of these approaches is the lack of clinical nurses’ Lack of necessary competencies, capabilities and opportunities for to effectively perform their educational role for nursing students. In this model, student clinical training is not absolutely academic and up-to-date. Additionally, the student’s learning needs are inconsistent with the clinical work needs of the organization, and sufficient support and supervision are not provided by the institutions [20–22]. In this category, the collaboration is between educational institutions and clinical nurses, and healthcare setting only cooperate in selecting nurses as clinical instructors. Clinical nurses are primarily in contact with nursing schools individually; moreover, there is often no structured link between the two educational and healthcare institutions. If there is a contract, it is between the nurse and the educational institution, and the healthcare institution and the clinical instructor do not receive adequate services from the educational institution. Some teaching models that fall into this category are Preceptorship, Internship, and Clinical Teaching Associate (CTA) [23, 24].

The second category which was developed to compensate for the shortcomings of the first category, is the academic-practice partnership pattern [20], which attempts to establish a two-way interaction between the academic and healthcare institutions [25]. This approach includes Joint Appointments, the Clinical Scholarship Model, and ، the Dedicated Education Unit (DEU) [16].

However, a literature review indicated that despite the relative improvements of nursing education in some aspects, these models, can’t provide the goals of the educational trustees. Also, In the models of this category, how the two educational and healthcare institutions collaborate, how the decision-making is shared, how the collaborative programs are supervised, as well as the job descriptions of the nurses and the nursing faculty members’ roles are unclear. (As faculty members) [17, 26, 27].

In Iran, since 2016, in response to the theory-practice gap, and in order to improve the quality of clinical teaching, recognition of the nursing faculty member in the clinical environment, and improvement in the quality of nursing care, Recruitment regulation Clinical Faculty Members was designed and was notified by the Ministry of Health and Medical Education to create a link between educational institutions and healthcare settings [28]. This regulation had some problems such as vagueness of the clinical faculty member’s role in reducing the theory-practice gap, the obscurity of the interaction and participation of academic and healthcare settings, and lack of clear strategies for implementing of regulation and achievement to the goals. Therefore, this regulation did not significantly change [29]. This issue was raised at the Supreme Council of Nursing Policy in the Ministry of Health and Medical Education, and it was decided that necessary measures be taken in this regard. These activities led to the design of Attending Nursing Teacher Project to develop the role of Clinical Faculty Member. This project was in line with the policies of the Deputy Minister of Nursing in the Ministry of Health and Medical Education, Board of Nursing Examinations and Evaluation, the Supreme Council of Nursing Policy It led to the introduction of the TPSN model (Teacher, Patient, Student, Nurse) within the Attending Nursing teachers Project framework, which is known as the Nursing Attending in the Iranian scientific community. This project aimed to alleviate the deficiencies of previous methods mentioned in academic-practice partnership models and eliminate the shortcomings of Recruitment regulation of Clinical Faculty Members by presenting a comprehensive model. The purpose of this study is to introduce and describe the innovative TPSN model with the aim to reduce the theory-practice gap.

## Methods

### Design of the study

This model is the result of a national project that has been carried out with the methodology of practical or collaborative action research. Research Action Cycles This study has four phases including problem identification, planning, and action and reflecting. In the problem identification phase, the challenges and solutions placing Clinical Faculty Members and its exit strategies were identified from the perspective of the clinical faculty members. In the planning stage, based on the suggestions and priorities of the participants and the available facilities and conducting focus group discussions, the initial conceptual model and framework was developed and then, based on it, the action plan was developed. In the implementation phase, the designed program was implemented with the help of participants at a clinical unit. In the reflection step, the strengths and weaknesses, the effectiveness of the program, the issues and problems of program implementation evaluated and strategies and methods to improve and facilitating the next cycle of action were decided.

### Study settings and participants

The research setting was the internal department of the Educational and Medical Center of Tabriz University of Medical Sciences. This ward provided services to female patients with cardiovascular disease. The first cycle was started in January 2019 and ended in July 2021. Participants in this study included clinical faculty members (*n* = 12), all ward nurses, Metron and two educational supervisors, all 7th semester nursing students (*n* = 45), MSc nursing student (*n* = 10), PhD students (*n* = 5) and Faculty educational administrators. The head of the department was a physician invited in the first meeting to get familiar to the project and to facilitate the communication between the research team and medical team.

### Data collection

Qualitative and quantitative data were collected and analyzed simultaneously.

Qualitative data: Interviews, focus groups and observations were used for data collection. The interviews were semi-structured and were held in a room in the department. In the beginning of the study, the interview started with statements such as: Please describe 1 day of your work. “Given your work experience, what are the barriers and challenges of establishing a nursing faculty in a clinical?” “What strategies do you suggest for developing the role of faculty members in the clinical?” Exploratory questions such as “Can you explain this with a real example” and “Can you make this a little clearer” were asked during the interview. Data collection continued until no new data was identified (data saturation). There were 10 focus groups and 25 meetings during the study period. The first focus groups were about the analysis of the interview and problem identification. Later, focus groups were held to decide about the possible interventions, to discuss the effects of interventions, and to solve the practical problems. All interviews and meetings were audio-taped and transcribed.

Quantitative data: Three questionnaires were completed at the beginning of the study in January 2019 and at the end of the study in July 2021. Clinical Learning Environment Inventory by nursing students (*N* = 60), job satisfaction in nursing questionnaire by all ward nurses (*N* = 15) and patient satisfaction with nursing care quality questionnaire by patients (*N* = 250) were completed.

Clinical Learning Environment Inventory (CLEI): This inventory has developed by Chan et al. in 2001. It has 42 items and six dimensions including Personalization, student satisfaction, involvement, individualization, task orientation and innovation. The items have a 5-point Likert scale from “strongly agree” to “strongly disagree”. Cronbach’s α coefficient of this questionnaire has been reported to be 0.88 [30]. This questionnaire has been translated to Persian and has good validity and reliability [31]. Cronbach’s α coefficient of this questionnaire in the current study was 0.8.

Job Satisfaction in Nursing Instrument: This questionnaire has been developed by Murrells et al. in 2005, has 20 items and six dimensions including the nature of work, development, relationships, education, work life interface and resources. The items have a 5-point Likert scale from “strongly agree” to “strongly disagree”. Cronbach’s α coefficient of this questionnaire has been reported to be 0.81 [32]. This questionnaire was translated to Persian. Its Cronbach’s α coefficient in this study was 0.78.

Patient Satisfaction with Nursing Care Quality Questionnaire: This questionnaire developed by Lascbinger et al. in 2005. Has 23 items. The items have a 5-point Likert scale from “satisfied very” to “very unsatisfied”. Cronbach’s α coefficient of this questionnaire has been reported to be 0.98 [33]. This questionnaire was translated to Persian and has a good level of reliability and validity [34]. It’s Cronbach’s α coefficient in this study was 0.86.

### Data analysis

Qualitative content analysis suggested by Graneheim and Lundman (2004) was used for the qualitative data analysis [35]. The MAXQD software version 10 software was used to manage the data. The content of the interviews was completely transcribed. To get a general idea, the transcripts were read by all authors several times. Then. The text about the participants’ experiences, problem identification, and suggested interventions were extracted, and subcategories and categories were discussed by all authors until consensus was reached.

The variables about the student perception of clinical learning environment, job satisfaction in nurses, and patient satisfaction with the quality of nursing care were presented descriptively by frequency, mean and standard deviation (SD) and the differences of the variables from the beginning and the end of the study were analysed using statistical tests including paired t test and independent t test. The data were analysed using SPSS software version 21. *P*-value < 0.05 was accepted as statistically significant.

## Results

### Problem identification

Qualitative data: there were 15 interviews with clinical faculty members. The Interviews were between 40 and 85 minutes with mean duration of 58 ± 15 minutes. Four focus groups were held. Six field notes were also recorded. After analyzing the qualitative data, the main problems were identified (Table 1):

**Table 1:** Categories and sub-categories derived from qualitative data analysis

Categories	Sub-categories
Lack of infrastructure and resources in the faculty	Shortage of human resources
	Deficiencies in educational structure and planning
	Lack of opportunities for faculty growth and prosperity
Separated nursing faculty in the care system	Lack of participation of faculty members in patient care
	Lack of integration of educational and care roles in faculty members
	Neglecting the caring role of faculty members in the nursing system
Lack of authority of a faculty member in care center	Lack of legal status and authority
	Lack of power to change
Lack of integrated and team system in clinical education and care	Negative attitude of physicians towards nurses
	Lack of effective professional interaction between nurses and faculty members
Barriers to playing the roles of clinical nursing faculty members	Role ambiguity
	Ambiguity in how to monitor and evaluate faculty performance
	Role pressure
An unpleasant experience for clinical faculty members with being placed in care centers	Feeling rejected from university
	Feelings of worthlessness
Non-participatory education and treatment system	Ineffective interaction between health care centers and the faculty
	Performance mismatch between faculty members and nurses

#### Lack of infrastructure and resources in the faculty

Shortage of human resources, Deficiencies in educational structure and planning, and lack of opportunities for faculty growth and prosperity, were the sub categories of this problem. A nursing faculty member said, *“In unity there is strength. I can eventually upgrade one or two departments and empower their nurses to carry out the nursing process, provided I have the necessary power and authority.”* Another participant said: *“Our most important problem is the educational structure, Clinical education of students is done in a traditional way. The nursing instructor has to teach seven to nine students in the ward. In addition, he has to play many roles in the clinic, such as attending hospital committees and training sessions, holding training courses for nurses, which is practically impossible.”*

#### Separated nursing faculty in the care system

Lack of participation of faculty members in patient care, lack of integration of educational and caring roles in faculty members and Neglecting the caring role of faculty members in the nursing system were the main problems mentioned by participants. A clinical faculty member said: *“In the nursing system of our country, the participation of the faculty in the care and treatment of the patient is a neglected circle. What the faculty wants from clinical faculty members is more of an educational and coaching role. Although clinical faculty members sometimes hold workshops at the hospital, they provide training and counseling for nurses. But these roles are not bold. In other words, the ultimate goal of improving the quality of patient care does not occur”.*

#### Lack of authority of a faculty member in the clinical

Lack of legal status and authority and lack of power to make changes were the main problems mentioned by clinical faculty members. One of the participants said: *“Clinical faculty have no place in care centers. At the hospital, I’m not part of this clinical system. I feel like a guest. As a coach, most of my energy is spent finding a place in the clinical wards to be accepted in the department and to be able to teach students”.*

#### Lack of integrated and team system in clinical education and care

Physicians’ negative attitude towards nurses and lack of effective professional interaction between nurses and faculty members were the sub categories of this problem. One of the participants said: *“Lack of inter professional collaboration is a major challenge in developing clinical faculty roles. There is no effective interaction between physicians and nurses or nursing faculty members with nurses, either in care or in training programs”.*

#### Barriers to playing the roles of clinical nursing faculty members

Role ambiguity, Ambiguity in how to monitor and evaluate faculty performance and Role pressure were the main problems mentioned by participants. One of the participants said: *“I do not understand the purpose of placing faculty members in the clinical. Is the purpose to improve the quality of clinical education? Or is the purpose to improve the quality of nursing care? The main purpose is missing”.* Another participant said, “I am now in charge of educational accreditation, continuing education, and the university-hospital interface, and I am required to attend all accreditation sessions. Most of the time, I have to leave students in the ward to attend corporate sessions. During the hospital sessions, the students of the ward regularly call my mobile phone.”

*An unpleasant experience for clinical faculty members with being placed in care centers:* Feeling rejected from university and Feelings of worthlessness were the main problems mentioned by clinical faculty members. One of the participants said: *“I’m really rejected by the college. This is my inner feeling. If they really do not want us, tell us clearly.”*

*Non-participatory education and treatment system:* Ineffective interaction between health care centers and the faculty and Performance mismatch between faculty members and nurses were the sub categories of this problem. One of the participants said: *“The partnership between the hospital and the faculty is not good. There seems to be an interaction between the two systems. There is no coordination between the hospital and the faculty on how we work.”* another participant said: “If the college specified your duties, they would *have to pay your monthly salary as well. Now that your monthly salary is paid by the hospital, we will determine your job description. You can also go to college to teach if we allow.”*

Quantitative data: clinical learning environment inventory by nursing students, Nursing Job Satisfaction Questionnaire by all ward nurses, and patient satisfaction with nursing care quality questionnaire by patients were completed. The score of perception of clinical learning environment was 14.06 ± 8.06 that shows moderate positive perception. The score of job satisfaction in nurses was 64.23 ± 5.98 that shows moderate satisfaction. The score of patient satisfaction with the quality of nursing care was 68.36 ± 5.41 that showed high satisfaction.

### Planning and action

In five focus groups, the problems and possible changes were discussed with nursing teachers, nurses, PhD students in nursing, MSc nursing students, nursing students, nursing managers. The suggestions were analysed and the feasibility of the plans was reviewed. In the 9th meetings of the research team and the panel consisting of 10 nursing experts, based on the literature review and the results obtained from the focus group meetings and individual interviews, the TPSN model was designed, which prepared the philosophical and theoretical basis of the work. The components of the model are as follows:

This model was designed based on the review of the literature, qualitative interviews with educational staff, including nursing professors, nurses, nursing Ph.D., master’s, and bachelor’s students, nursing managers, and expressed views in focus group discussions.

The academic-practice integration Model of TPSN, which was developed within the Attending Nursing teachers Project, is a dynamic and interactive model for accountability in nursing and similar settings and disciplines. Unlike the Medical Education model that includes patients, students, and physicians as the three points of a triangle, this model, which is shaped like a large triangle, places the person in need of care and treatment (patient, client, family, or society) in the center of the triangle, aiming to focus on the healthcare receiver.

The model consists of three components (three small triangles). In Iran, as in other countries, it is impossible to turn all nurses into nursing faculty members in Medical Sciences Universities and teaching hospitals affiliated with them due to various factors such as inadequate with Master and PhD degree in nursing and few organizational positions for faculty members in nursing schools. Therefore, in this model is attempted to manage this issue with logical solutions. The constituent members of this model include the officials of the School of Nursing and Midwifery (head, deputy for education and postgraduate studies, department manager, and deputy of administrative and financial affairs), resident faculty members in the clinical setting, undergraduate and postgraduate nursing students, head nurses, clinical nurses, a, educational supervisors, nursing manager, and dean of the hospital.

It should be noted that the job descriptions and roles of all this members, their performance evaluation and educational materials were provided and reviewed by an expert panel. Afterward, the necessary changes were made and re-approved by the expert panel.

**Fig. 1:**
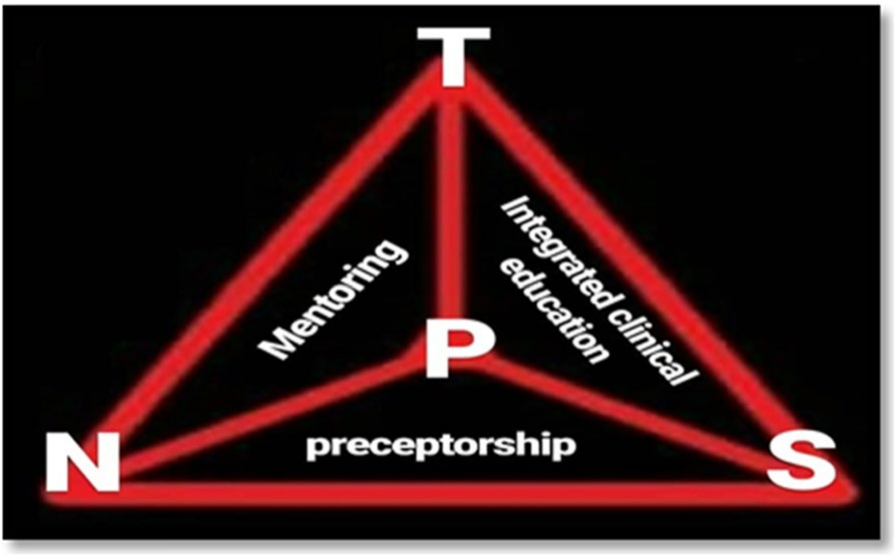
TPSN Model (Teacher, Patient, Student, Nurse, and others): Academic-Practice Integration

In this model, the large triangle consists of three components (smaller triangles) (Fig. 1):

- Mentoring component (TPN triangle)
- Preceptorship component (NPS triangle)
- Integrated clinical education component (TPS triangle)

### Mentoring component (TPN triangle)

**Fig. 2:**
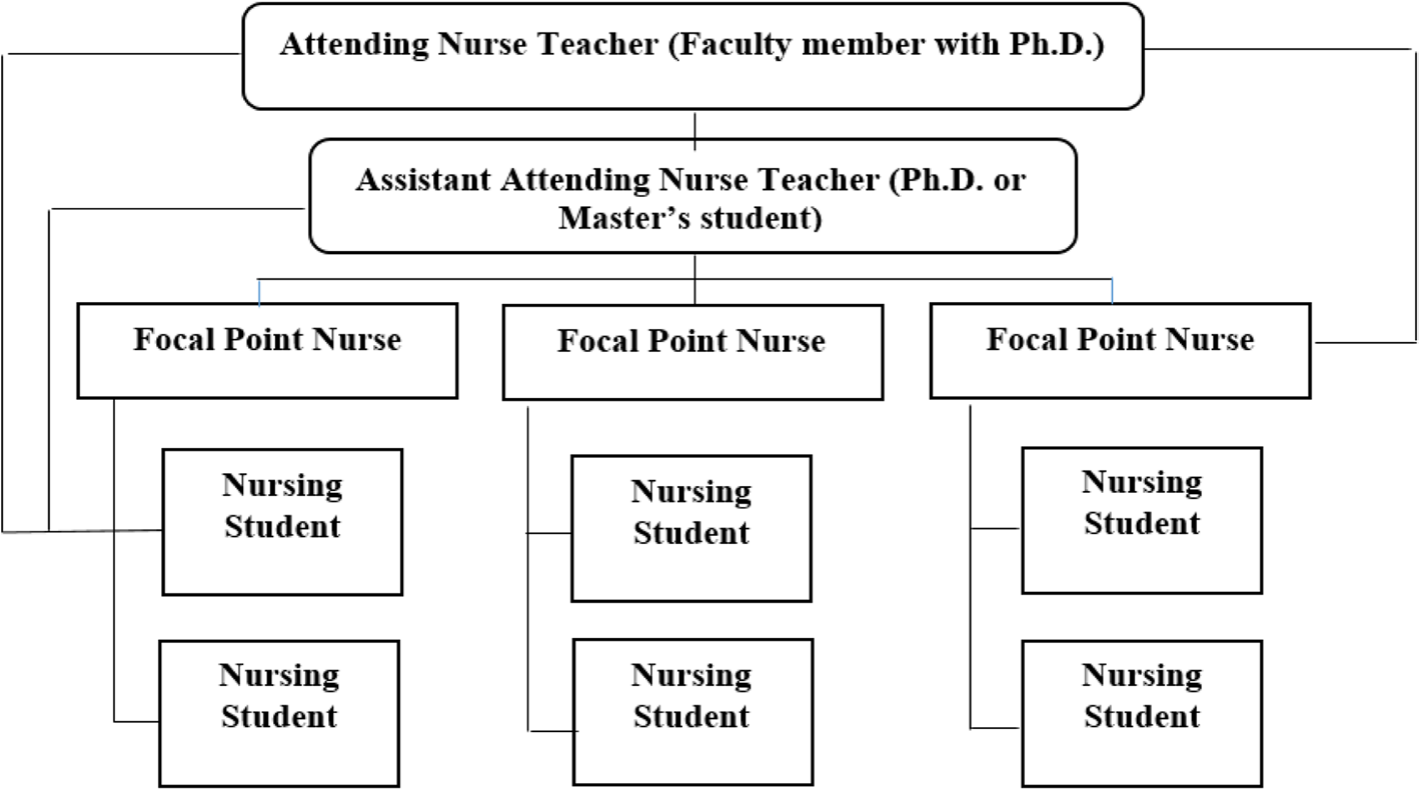
Mentoring component

The mentoring component (TPN triangle) is formed by the interaction between patients, nurse and nursing teachers (clinical faculty members). This triangle aims to show the effect of faculty members on health and nurses. Resident nursing teachers fulfill their responsibilities through specific strategies introduced in this triangle, such as implementing nursing grand rounds, morning rounds, journal club, patient visits by attending nurse teacher, and educational rounds. Additionally, they apply required changes in the ward in terms of providing of evidence base cares, empower nurses to provide nursing care based on the nursing process and create educational proper environment for learning and teaching. These activities are done with the participation of nurses and, in some cases, nursing students (Fig. 2).

### Preceptorship component (NPS triangle)

**Fig. 3:**
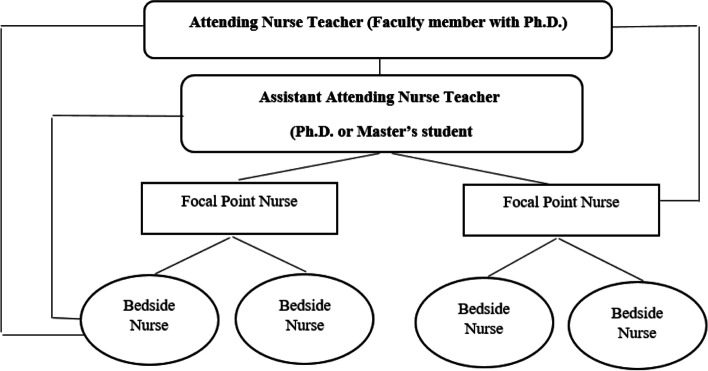
Preceptorship component

The preceptorship component (PSN triangle) is formed through nurses’ and other health service providers’ interaction with students and patients. Its structure is based on the adjusted preceptorship model, in which nurses, after nursing teachers empower them in the mentoring triangle, can play a suitable role in nursing students’ education as role models. In this model, nurse preceptors become empowered by mentoring due to the faculty members’ presence in the clinical setting, and this empowerment exists in both educational and healthcare settings (Fig. 3).

### Clinical education integration component (TPS triangle)

**Fig. 4:**
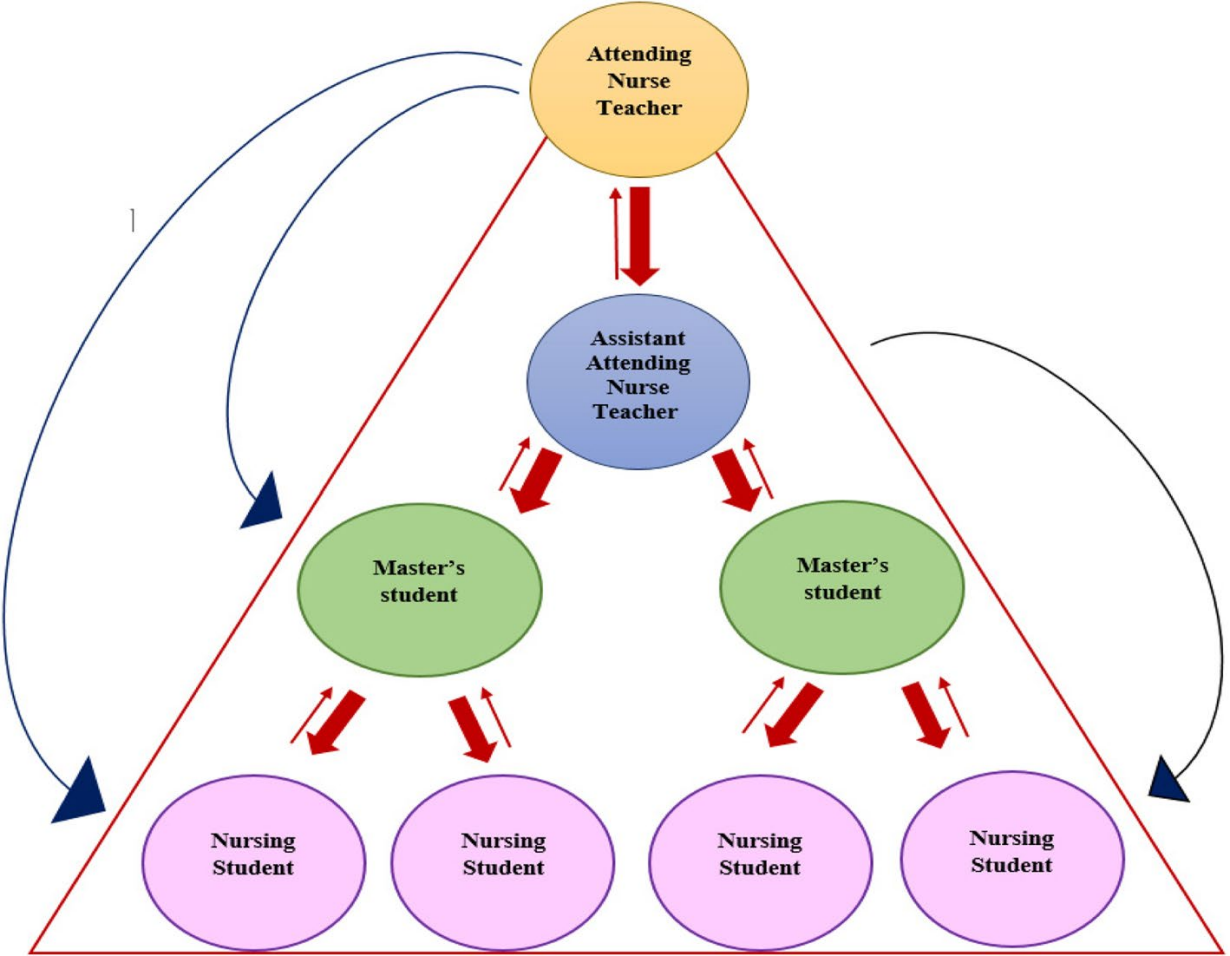
Integrated clinical education component

In the clinical education integration component, using specific strategies such as cascading education, nursing grand rounds, journal clubs, and education based on the nursing process, etc. undergraduate and postgraduate nursing students are trained and empowered in a dynamic and interactive system with defined specific job descriptions for students, head nurses, and trained clinical nurses. An organizational memorandum of understanding is also concluded between the school and the teaching hospital (Fig. 4).

### The relationship between the components of this model

**Fig. 5:**
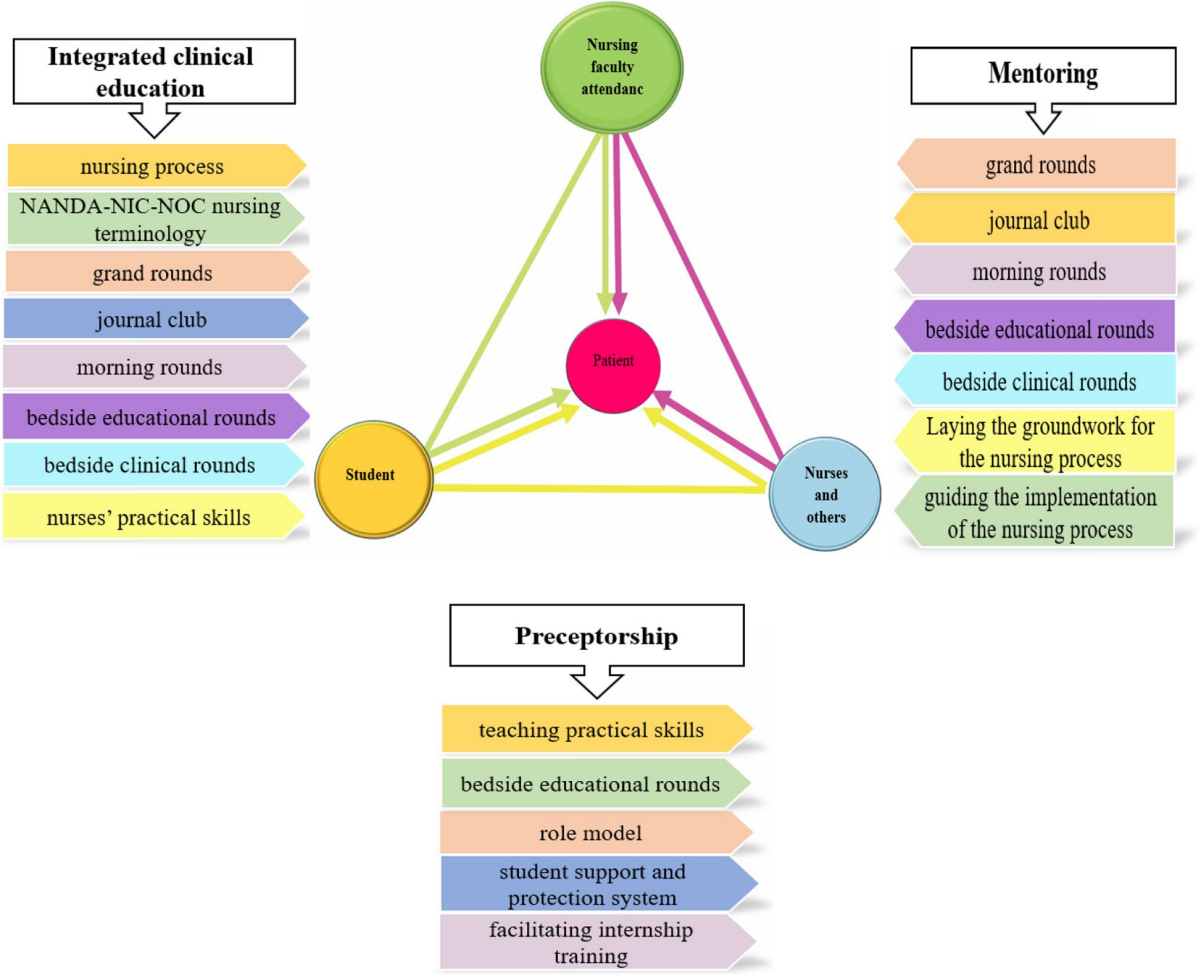
The description of the TPSN Model

Each of the components of this model alone will not be able to eliminate the ultimate goal of bridging the theory-practice gap. As the first component, the mentoring component focuses on the influence of faculty members on the fields of health and nurses and empowers nurses to provide nursing care based on the nursing process and create an educational environment. The second component is preceptorship, in which nurses, after empowerment by the mentoring component, work as preceptors in the clinical education of students. In this component, students of different levels are empowered in a dynamic and interactive system defined with specific job descriptions using specific strategies such as cascading education, nursing grand rounds, education based on the nursing process, and the educational curriculum (Fig. 5).

### Development and finalization of the TPSN model

The implementation of nursing education based on the TPSN model was discussed and approved during joint sessions between the nursing school and the teaching hospital in Tabriz, Iran, with the presence of the deputy health minister for the nursing department. Furthermore, necessary spaces for clinical faculty members’ and Ph.D. students’ residence were provided and equipped.

The women’s cardiology ward of the teaching Hospital in Tabriz was selected to conduct the pilot study of the model. This ward had 30 beds and 15 nurses in all shifts. Patients hospitalized in this ward were diagnosed with cardiovascular disorders such as angina pectoris and myocardial infarction. All 15 nurses working in the ward were included in the study. A briefing session was held for students at the beginning of the internship to discuss rules and regulations, expected goals and outcomes, vision and goals, members participating in education and their roles, students’ expectations and job descriptions, and interaction with team members (attending nurse teacher, other student in the cascaded education, and preceptors). The schedule of medical-surgical nursing students of different levels was prepared with the cooperation of the researchers of the study to be implemented in 2019. The department chair, deputy for education, nursing faculty members, the dean of the nursing school, the head of the teaching hospital, the hospital deputy for education, the head of nursing services, the head nurse of the ward, and students were informed. Monitoring the nurses was done by the head of nursing services, the nursing educational supervisor, and the head nurse. During the interview, oral feedback was obtained from the students and discussed and analyzed after being summarized by the research team.

In the model mentioned above, the clinical faculty members were present full time in the cardiology ward, and the nursing students of different levels attended for education in rotation. The faculty members in the ward provided education to the students and nurses. Education and practice were done within the framework of the nursing process. Theoretical and practical training workshops on the nursing process were held in groups in 4 two-hour sessions. In order to establish clinical and authentic learning, one of Ph.D. nursing students trained the nurses working in this ward individually for 6 months using this tool under the supervision of a resident nursing teacher. In order to improve the utilization of the nursing process, daily rounds were reviewed and discussed by the nursing teacher residing in the ward to review the nursing process performed by the nurses in different shifts. During the rounds and different shifts, Ph.D. and master’s students were present to train and advise nurses. Moreover, the attending nursing teacher held two grand round sessions and three journal clubs to update the knowledge of the nursing staff (standards, guidelines, instructions, etc.) and to plan for the development of evidence-based practice for the nurses of the relevant ward under the supervision of the dean of school in coordination with the educational and nursing management supervisor of the hospital.

## Reflection

During action research and in the end of the program, the reflections were gathered through focus groups and interviews. Some changes were made in the planning according to the reflections. Nursing process workshops and training booklets tailored to the ward for nurses’ empowerment were added to the programs. Overall, most of the reflections were very positive. For example, one participant stated that these changes have developed a holistic view of patient care in students and nurses. In addition to physical dimensions, nurses considered other dimensions such as psychological and spiritual and cultural factors in the patient’s examination. Based on the problems identified in the patient, they designed and implemented nursing diagnoses and care plans for patients. “The interaction between the faculty and the clinical nurses also improved.” One of the participants said: “Club journals have improved the relationship between the School of Nursing and nurses in clinical settings, and this is a very important aspect.” Another positive reflection of the model implementation was the role and position of clinical faculty members. In this regard, one of the participants said: “We always hear from nurses that doctoral nurses are only for student education and they have always been separated from practice. But this model has been able to define the role and position of a nursing doctor in the clinical.”

This model has been evaluated and discussed in several scientific conferences, especially in the national symposium titled “Introduction and Criticism of Educational Innovations in Nursing,” and “Responsive Medical Education Conference” [36, 37] Furthermore, a national webinar titled “Attending nurse teacher project: based on TPSN Model” was held with members of the Board of Nursing Examinations and Evaluation, heads, and deputies for the education from nursing and midwifery schools throughout the country [38]. The results, feedback, and experiences of the implementation of the model were presented, and the model was analyzed.

In the end of the action research, participants completed the questionnaires once again. The score of Students’ perception of clinical learning environment was increased to 142.26 (SD = 8.06) from 102.56 (SD = 8.05) that showed a statistically significant difference (*p* = 0.0001). the score of job satisfaction in nurses was also increased to 64.23 (SD = 5.98) that showed a statistically significant difference (*p* = 0.0001). Patient satisfaction with the quality of nursing care was 41.52 (SD = 6.03) that increased to 68.36 (SD = 5.41) (*p* = 0.0001) (Table 2).

**Table 2:** Perception of clinical learning environment, Nursing Job Satisfaction and patient satisfaction with nursing care quality before and after first cycle of the participatory action research

Variables	Before the intervention (Mean ± SD)	After the intervention (Mean ± SD)	***P***-Value
Perception of Clinical Learning Environment	102.56 ± 8.05	142.26 ± 8.06	.0001
job satisfaction in nursing	43.21 ± 3.82	64.23 ± 5.98	.0001
Patient satisfaction with nursing care quality	41.52 ± 6.03	68.36 ± 5.41	.0001

## Discussion

The mismatch between theoretical education and the performance of nurses in the practice leads to the lack of use of theoretical knowledge and adherence to traditional methods in the practice. This prevents the development of theoretical sciences in nursing and leads to a decline in the quality of nursing care [5, 7]. This participatory action research study showed that the TPSN academic-practice integration model, by defining the role and position of clinical educators in clinical settings, defining the formal scope of their authority and responsibilities, along with the implementation of mentoring and preceptorship programs can lead to improved clinical education and quality of nursing care. What distinguishes the TPSN model from other approaches is its main focus on promoting nursing care for patients as key beneficiaries. In Iran, in recent years, the public and the government have criticized nurses because of poor quality of patient care, and the divergence between nursing theory and clinical practice has also been recognized by some Iranian nursing researchers [39].

Each of the components of this model alone will not be able to eliminate the ultimate goal of bridging the theory-practice gap. Studies also show that many strategies such as internship, mentorship and preceptorship programs have been applies to reduce this gap in many countries [40–42]. Although in some cases these programs have been to some extent successful, the subject still one of the major issue in nursing education which needs further research [43].

In this study, the mentoring component as the first component of the model, in order to influence the clinical faculty member on health and nurses. Resident nursing teachers, along with the master’s and doctoral students in nursing, fulfill their responsibilities through special strategies introduce in this triangle, such as implementing nursing grand rounds, morning rounds, journal club, patient visits by attending nurse teachers, and educational rounds. They also apply the required changes in the ward in terms of providing of evidence base cares, empower nurses to provide nursing care based on the nursing process and create educational proper environment for learning and teaching. Faculty members have a responsibility to empower nurses to have critical thinking, the power of change, and creative approaches to problem solving. The study by Chaghari et al. Showed that faculty members play a very important role in empowering nurses. In several studies, mentoring programs have been proposed to develop role, professional support, and effective interaction between nurses and faculty members [44–46].

The second component is the preceptorship that nurses play a role in the clinical education of students after nursing teachers empower them in the mentoring. Utilizing skilled and experienced clinical nurses under the supervision of clinical faculty members promotes student learning. In many studies, despite the usefulness of the Perspective program, many obstacles such as high workload, lack of educational competence of nurses, insufficient support from school administrators and care centers have been reported in the effectiveness of this program [47, 48]. According to research evidence, the main weakness of this method is the unpreparedness of nurses to effectively perform their educational role for nursing students. On the other hand, the student’s learning needs are not in line with the work needs of the organization, and sufficient support and supervision is not applied by the organization and the faculty to educate students [22, 48, 49]. According to the TPSN model, before clinical nurses act as preceptor, they are empowered by the clinical faculty members together with postgraduate and doctoral nursing students and play their role under the direct supervision of clinical faculty members.

In the clinical education integration component, using specific strategies such as cascading education, nursing grand rounds, journal clubs, and education based on the nursing process, etc. undergraduate and postgraduate nursing students are trained and empowered in a dynamic and interactive system with defined specific job descriptions for students, head nurses, and trained clinical nurses. In cascading education, top-down instruction flows from a qualified instructor to different levels of students [50]. In the TPSN model, for integrated clinical training, attending nurse teacher works with three levels of PhD, M.Sc. and BSc students in line with the needs of patient in order to identify problems based on Gordon’s model. Studies show that when students of different levels learn in an educational environment, in addition to immediate access to the teaching resource, their learning rate increases due to the existence of several teaching resources [51, 52]. It also allows coaching to be taught to postgraduate and doctoral students. Studies show that implementation grand rounds, journal clubs and clinical education based on the nursing process, holding clinical and educational rounds at bedside play a very important role in reducing the gap between theory and practice. It promotes critical thinking, clinical reasoning, and the application of theoretical learning to practice [53–55].

## Conclusion

A new and innovative model was proposed to reduce the theory-practice gap in the present study. This model increases the collaboration between educational institutions and healthcare setting compared with the previous models. The TPSN model helps students, nurses, and nursing instructors integrate theoretical knowledge with clinical practice and act as professional nurses. What distinguishes the TPSN model from other approaches is its main focus on promoting nursing care for patients as key beneficiaries. Moreover, this model provides a solid framework for developing higher education curricula and research in the future. However, more research is needed to ensure the positive outcomes of this model and evaluate its effectiveness and benefits for the main stakeholders. Implementation of nursing education based on this model requires strong policies and organizational support from all stakeholders. Moreover, for the correct implementation of the model, it is necessary to make preparations to empower students, professors, and nurses.

Healthcare and educational institutions should also consider the initial costs (structural facilities, space, and equipment) of implementing the model. Positive feedback was received regarding the academicization of the healthcare setting, increase in collaboration between faculty members, healthcare professionals (nurses, physicians, and others), and students, increase in the quality of nursing care, and increase in students’ learning opportunities, which will be reported in detail in future articles.

## Data Availability

The datasets used and/or analysed the current study are available from the corresponding author upon reasonable request.

## Abbreviations

TPSN: Teacher, Patient, Student, Nurse
CLEI: Clinical Learning Environment Inventory
SD: Standard deviation
